# The role of party preferences in explaining acceptance of freedom restrictions in a pandemic context: the Italian case

**DOI:** 10.1007/s11135-022-01436-3

**Published:** 2022-06-07

**Authors:** Riccardo Ladini, Nicola Maggini

**Affiliations:** 1 Department of Social and Political Sciences, University of Milan, Via Conservatorio, 7, 20122 Milan, Italy; 2 Department of Social and Political Sciences, University of Milan, Via Pace, 10 Third Floor, 20122 Milan, Italy

**Keywords:** Attitudes towards freedom restrictions, Covid-19, Party preferences, Institutional trust, Collectivism, Survey research

## Abstract

As a consequence of the Covid-19 pandemic, several governments adopted disease containment measures limiting individual freedom, especially freedom of movement. Our contribution aims at studying the role played by party preferences in explaining attitudes towards those freedom limitations during the pandemic, taking into account the moderating role played by confidence in institutions and collectivist-individualistic orientations. Focussing on Italy, as the first western democracy to be hit by Covid-19 and to adopt harsh restrictive measures, we analyse data coming from the ResPOnsE COVID-19 project. Our study initially investigates whether attitudes towards freedom restrictions are associated with the dynamics of the pandemic and the institutional responses to it. Then, through multilevel regression models, we test several hypotheses about the relationship between party preferences, confidence in institutions, collectivistic orientations and public acceptance of Covid-19 containment measures limiting individual freedom. Findings show that party preferences are associated with different attitudes towards freedom restrictions to contain the pandemic, but this occurs only if people have individualistic orientations. Collectivistic orientations and confidence in institutions are positively associated with acceptance of freedom restrictions, regardless of party preferences. As regards the latter, neither a classical ideological explanation (conservative people more inclined to accept limitations to personal freedoms) nor a government-opposition explanation (supporters of government parties more inclined to accept freedom restrictions) seems to be adequate to fully account for the mechanisms behind acceptance of Covid-19 harsh containment measures. Thus, we offer an alternative ideological explanation by pointing out the ambiguous nature of contemporary right-wing populisms.

**Supplementary Information:**

The online version contains supplementary material available at 10.1007/s11135-022-01436-3.

## Introduction

During the Covid-19 crisis, all democratic governments have introduced measures that limited citizens’ freedoms to counter the pandemic, although with different timing, tools and degrees of coercion (Cheibub et al. 2020; Stasavage 2020). If individual freedoms are usually limited in authoritarian systems, the same does not usually occur in democracies. Indeed, advanced democracies are so defined because their constitutions and laws guarantee protection for the fundamental freedoms of citizens. The need to curb the spread of the virus has therefore subjected contemporary democratic regimes to a very severe test and boosted a renewed attention in polity studies and political culture research on well-known issues of state operation, state resilience against a threat, public trust in institutions, citizens’ compliance with authorities’ decisions and differences in individual attitudes depending on political-ideological-cultural orientations.

Within the broad theme covered by the Special Issue on “Paradigmatic Insights in Polity Studies”, this article fits the political culture research area by investigating the relationship between political preferences, confidence in institutions, collectivistic-individualistic orientations and public acceptance of Covid-19 containment measures limiting individual freedom, especially freedom of movement and of meeting other people, in the Italian context. Italy, indeed, can be seen as a paradigmatic case to study these issues as it was the first western country to be hit by Covid-19 and to adopt harsh non-medical containment measures against the virus. Starting from March 9, 2020, during the most acute phase of the first wave of the pandemic, Italian authorities have been inflexible in implementing containment measures including the imposition of freedom restrictions (e.g. travelling, social meetings, gatherings, etc.) through a national lockdown aiming to “push down” the Covid-19 epidemic curve. Since then, for two months citizens could leave the house for only three reasons: proven work needs, health reasons and other situations of need. Schools, commercial activities (except for essential services like supermarkets) and most economic activities were closed. On May 4, 2020, the government started to relax the restrictions and on June 3 people were allowed to travel throughout the country and abroad. At the end of July 2020, Italy had counted a total amount of 245,000 infected cases, 35,000 deaths as well as 12,000 individuals still infected.

In this unprecedented global emergency context, it is therefore noteworthy to analyse Italians’ attitudes regarding the acceptance of freedom limitations. The health risks caused by the pandemic could have threatened the rising quest for emancipative values, such as freedom, highlighted in socio-political research on value change (Welzel 2013). Moreover, attitudes towards freedom limitations are supposed to depend on various social, health, and political factors. Indeed, during an exceptional event such as a pandemic individual attitudes on sensitive issues for democracy can be influenced both by the context itself (and its evolution) and by their *a priori* political and values’ orientations. In this latter respect, this article will investigate whether—and how—party preferences allow explaining attitudes towards freedom limitations during the exceptional pandemic period. As shown by previous research, ideological orientations matter when it comes to acceptance of restrictions on freedoms and obedience to authority in a situation of perceived threat, pinpointing the role played by traditional left–right orientations (McClosky 1964; Sullivan et al. 1982; Bialasiewicz and Eckes 2021). Moreover, attitudes towards freedom limitations during the Covid-19 crisis could be explained by non-partisan value orientations, such as collectivistic-individualistic orientations (Yang and Tsai 2020). Similarly, obedience to authority can be influenced by confidence towards institutions in general in the context of a large-scale threat to collective and personal security (Davis and Silver 2004), and more specifically during the Covid-19 pandemic (Guglielmi et al. 2020). Our contribution also analyses the moderating role played by both confidence in institutions and collectivistic-individualistic orientations in the relationship between party preferences and attitudes towards freedom limitations. Consequently, we aim to answer three main research questions, one exploratory and two hypothesis-driven, relying on original survey data coming from the ResPOnsE COVID-19 project^1^:

1. Did attitudes towards freedom limitations vary in the light of the evolution of the pandemic and the changing measures taken by the government? And how?
2. What is the role of party preferences in explaining the willingness to accept restrictions on individual freedom in the emergency context?
3. Is the effect of party preferences on such attitudes moderated by trust in institutions and collectivistic orientations? And how?

The article is structured as follows. In the next section, we present the theoretical framework of the research and the related hypotheses; we then discuss the data, measures and methods employed to test the hypotheses and in the subsequent section we present our results. Finally, we discuss the main implications of the empirical results and provide concluding remarks.

## Theoretical framework and hypotheses

Tackling a pandemic is an extremely demanding challenge for any political regime, both democratic and authoritarian. Among the initiatives implemented to contain the infections from Covid-19, over the last two years the governments of most countries of the world have imposed restrictions on some individual freedoms, which entail the democratic dilemma between public health and civil liberties. In this regard, scholars investigated the consequences of such decisions on public attitudes and examined how people responded to governments’ decisions, exploring the explanatory factors behind the different levels of acceptance of Covid-19 containment measures. As for the latter point, the attention has been put on the role played, among other things, by political orientations.

In particular, it is worth considering the influence of personality traits and ideological orientations that previous research suggests should structure people’s acceptance of illiberal policies in a situation of perceived threat, such as the context of terrorist attacks. In this regard, it has been highlighted the role played by authoritarianism (Altemeyer and Altemeyer 1996; Hetherington and Suhay 2011; Stenner 2005) and left–right ideological orientations. The latter are key factors to explain party competition and voting behaviour in the European context, especially in western countries. Indeed, the left–right dimension is widely recognised among citizens (Fuchs and Klingemann 1989; Knutsen 1995) as a heuristic to infer policy preferences of parties and thus to vote for the party that is closer on the same dimension (Downs 1957). Although in recent years the dimensionality of the political space has been questioned, with scholars stressing the emergence of new cultural dimensions (Kriesi et al. 2006; Hooghe and Marks 2018) of political conflict beyond the economic left–right dimension rooted in the class cleavage (Lipset and Rokkan 1967), it is important to note that the left–right concept is a dynamic communication device subject to social negotiation (Laponce 1981; Fuchs and Klingemann 1989; Knutsen 1995) and over time it has proved able to absorb new meanings (Flanagan 1982). According to the classical “progressive-conservative” antithesis (Middendorp 1978), economically and culturally progressive stances can be separated from economically and culturally conservative ones: economic equality and cultural pluralism on the left and economic freedom and cultural uniformity on the right. In this broad sense, the terms “left–right” (or “progressive-conservative”) have a meaning similar to that of “liberal-conservative” in the US context, especially as for cultural stances. For the purposes of our research, it is worth mentioning that previous studies, generally referred to the US, found that in a situation of perceived threat individuals with more conservative positions usually show greater obedience to authority (McClosky 1964; Sullivan et al. 1982) and tend to view rights as more situational and contingent (McClosky and Brill 1983). Conversely, progressive people (liberals in the US) tend to think of rights as natural and inalienable (McClosky and Brill 1983). Davis and Silver (2004), while analysing people’s willingness to trade off civil liberties for greater personal safety and security after the 9/11 terrorist attack in the US, found similar patterns, by arguing that conservatives are more concerned for law and order over civil liberties. For what concerns the Covid-19 pandemic situation, Wnuk et al. (2020) showed that attitudes towards Covid-19 tracking technologies, which could be intended as possible violations of individual freedom, were more favourable among people with right-wing authoritarian views and high moral conservatism. Following these theoretical arguments and considering that the Covid-19 pandemic can be definitely considered as an emergency situation of perceived threat, we can expect that:

### H1a

Individuals preferring right-wing parties are more likely to accept restrictions on freedom imposed by public authorities to contain the pandemic.

However, this hypothesis could be undermined by the fact that during the Covid-19 crisis, “individual sovereignty” is pitted against the sovereign power of states by an ideological heterogeneous coalition (anarchists, libertarians, anti-vaccine conspiracists, right-wing nativists), whose lowest common denominator is the anti-establishment position (Bialasiewicz and Eckes 2021). Furthermore, according to a well-established stream of research (Campbell et al. 1960; Cohen 2003), partisan identification is a key factor in shaping people’s attitudes about public policies. In this regard, Arceneaux et al. (2020) conducted a series of experiments in the US and UK during the first wave of the Covid-19 pandemic finding that citizens are more likely to accept limitations on individual freedoms if they are championed by politicians of their preferred political party, although there is an overall tendency to resist policy proposals that include clear violations of constitutional rights. In this regard, given that restrictive measures are supported by government parties, we can hypothesise that:

### H1b

Individuals preferring opposition parties would be less likely to accept restrictions on freedom imposed by public authorities to contain the pandemic.

It is obvious that the two competing hypotheses about the relationship between party preferences and attitudes towards freedom restrictions cannot be disentangled if right-wing parties are in power and left-wing parties are in opposition. However, in the Italian context, the *ideological explanation* and the *government-opposition explanation* cannot overlap because the national lockdown was implemented during the first wave of the pandemic under the cabinet of Giuseppe Conte, when centre-right parties were in opposition. Indeed, the main parties representing the opposition in Parliament were Go Italy (*Forza Italia*, FI), the League (*Lega*) and Brothers of Italy (*Fratelli d’Italia*, FdI): the former is a mainstream centre-right party, whereas the latter two are right-wing populist parties. Conversely, the main parties supporting the government were the Five Star Movement (*Movimento 5 Stelle*, M5s—a populist post-ideological party) and the Democratic Party (*Partito Democratico*, Pd—a mainstream centre-left party). The government-opposition hypothesis can be further tested longitudinally in light of the change in the Italian government that occurred in February 2021, with the birth of a national unity cabinet led by Mario Draghi. By considering that two centre-right parties (Go Italy and the League) moved from the opposition to the government, it is possible to test whether in 2021 the attitudes of individuals supporting those parties tend to converge towards attitudes of people supporting other government parties.

Although important, party preferences are not the only factor that can have an impact on attitudes towards freedom restrictions imposed by public authorities to contain the pandemic. Scholars referring to the institutional theory (Baumol and Blinder 2008) have stressed the key role of confidence in institutions in explaining the varying degrees of citizens’ compliance with political measures or duties. As shown by research on public opinion attitudes in the context of a large-scale threat to collective and personal security (Davis and Silver 2004), it is plausible to hypothesise that people who show greater obedience to the provisions of the authority during a pandemic are those with the highest levels of trust in the institutions that those provisions adopt and implement. This also applies to the public acceptability of non-medical measures to limit the spread of infectious diseases, such as Ebola (Vinck et al. 2019), SARS (Tang and Wong 2003) and H1N1 (Prati et al. 2011). As regards Covid-19, empirical evidence of this relationship is found both at the aggregate (Barrios and Hochberg 2020; Bargain and Aminjonov 2020) and individual level (Han et al. 2021), although at the individual level results are mixed (Dohle et al. 2020; Jørgensen et al. 2021). In this regard, Guglielmi et al. (2020), focussing on the Italian case, provide an empirical test of the different mechanisms that can produce a different sign of the relationship between confidence in institutions and adherence to preventive measures against the pandemic. Although these mixed results, we can argue that attitudes towards freedom restrictions during the first wave of the pandemic are best explained by the so-called “cascade of confidence” mechanism (Guglielmi et al. 2020), according to which confidence in institutions is positively associated with adherence to the Covid-19 restrictive measures enacted by trusted public institutions. Consequently, we hypothesise that:

### H2

Individuals with high confidence in institutions are more likely to accept restrictions on freedom imposed by public authorities to contain the pandemic.

Apart from confidence in institutions, there is another factor that can have a strong impact on the attitudes towards freedom restrictions during the pandemic: predispositions about the collective and the individual. In this regard, political culture studies on East Asian countries show how these countries are considered to emphasize the value of obedience and conformity because of their cultural legacy rooted in Confucianism (Shi 2015; Zhai 2018, 2019), pointing out also at the individual level the positive relationship between Confucian values and political confidence (Shin 2013). Among these values, there is the idea that the collective interest of the national community prevails over the interests of the individual. As argued by Van Tubergen (2020, p. 432), indeed, “when people have collectivistic values, loyalty to the group is regarded as of utmost importance and individuals are supposed to consider group interest first and foremost”. During the Covid-19 pandemic, and especially during its most acute phase, the group interest was intended in prioritizing obedience to authorities over individual freedom. In this regard, Yang (2020) showed how public acceptance and compliance with the harsh measures taken by the Taiwanese government was because of the cultural legacy of Taiwanese people prioritizing the collective over the individual. Similarly, Yang and Tsai (2020) found that Taiwanese people with higher social trust were more likely to exchange their civil liberties with public safety, although people who support democratic values and pursue collective security tend to avoid violating privacy. Also, Frey et al. (2020) found that individualistic cultural traits of Western Europeans and North Americans are associated with negative attitudes towards government interventions (Frey et al. 2020). Although individualistic values are more widespread in Western societies (Welzel 2013), existential insecurity generally leads to an increase of collectivistic values (Inglehart and Welzel 2005) and this could have occurred also during the pandemic. At the individual level, collectivistic orientations are not necessarily politicised, in the sense that could not coincide strictly with authoritarian orientations and might cross-cut the left–right divide. In this respect, we can expect that these orientations play a role at the time of the pandemic in explaining a greater willingness to accept freedom restrictions than we would expect from purely political factors such as party preferences or long-term support such as trust in institutions. Hence, we can expect that:

### H3

Individuals with a collectivistic orientation are more likely to accept restrictions on freedom imposed by public authorities to contain the pandemic.

So far, we have developed two rival hypotheses about the role of party preferences in explaining different attitudes towards freedom restrictions during the pandemic and we have pointed out the role confidence in institutions and collectivistic orientations could play. A final point worth investigating is the interplay between these two latter attitudes and party preferences in shaping attitudes towards restrictions on freedom imposed by public authorities to contain the pandemic. As regards the interplay between partisanship and confidence in institutions within the pandemic context, Vezzoni et al. (2022) showed that in Italy differences in beliefs in alternative accounts on the origin of the virus among people with different party preferences are lower when they are highly confident in institutions.

Analogously, we can expect a similar pattern when party preferences interact with institutional trust—and collectivistic values—in influencing attitudes towards freedom limitations in the same pandemic context. Relying on the literature we have discussed so far, we assume that confidence in institutions and collectivistic orientations are strong predictors of opinions on limitations to individual freedoms to counteract Covid-19 diffusion and such attitudes are likely to be boosted when there is a widespread perception of collective threat as during a pandemic because of the so-called “rally ‘round the flag” effect (Mueller 1970). The latter describes citizens’ increased support for national governments after a terrorist attack or during an international crisis. As noted by various scholars (Bol et al. 2020; De Vries et al. 2020; Schraff 2020), democratic institutions benefitted from a sort of this effect after Covid-19 outbreak, especially during the first phase of the pandemic. Similarly, we expect that trust in institutions and collectivism increase at the aggregate level to such an extent as to cross-cut party preferences, meaning that several supporters of parties of different type (left-wing or right-wing, government or opposition) are characterised by stronger collectivistic orientations and higher confidence in institutions and thus similarly accept freedom restrictions to counteract Covid-19 spread. Consequently, at the individual level, we can expect that confidence in institutions and collectivistic orientations moderate the relationship between party preferences and attitudes towards freedom restrictions. In other words, we hypothesise that the effect of party preferences on attitudes towards freedom restrictions is conditioned by the level of confidence in institutions and the strength of collectivistic orientations: when people have high confidence in institutions and/or strong collectivistic orientations, party preferences are supposed to lose their explanatory power. Hence, our two conditional hypotheses read as follows:

### H4

Party preferences are more strongly associated with attitudes towards freedom restrictions among people with lower confidence in institutions.

### H5

Party preferences are more strongly associated with attitudes towards freedom restrictions among people with individualistic orientations.

## Data, measures and methods

### Data

To test our research hypotheses we employed ResPOnsE COVID-19 survey data, collected during the pandemic by the SPS TREND Lab of the University of Milan (Vezzoni et al. 2020).

The first wave of the survey was carried out during the first phase of the pandemic in Italy, from April 6 to July 8, 2020. The survey adopted a rolling-cross-section design which allows analysing the dynamics of public opinion over the period of observation, as the day of invitation for participating in the survey is randomly assigned. The sample was drawn from an opt-in panel of an Italian survey research institute (Swg S.P.A.) and reproduces population distributions for sex and geographical area of residence. Overall, 15,775 Italians were CAWI (Computer Assisted Web Interviewing) interviewed on behaviour compliance and several political and social attitudes and opinions, either specific to Covid-19 pandemic or more general (for more detailed information on the first wave of ResPOnsE COVID-19 survey, see Vezzoni et al. 2020; Biolcati et al. 2021). On average, the first wave of the survey collected about 165 daily interviews, yielding a total of over 1000 interviews per week. About one year later, during the third phase of the pandemic in Italy, a following wave of the survey was carried out from March 17 to June 16, 2021. Analogously to the first wave, it adopted a rolling-cross-section design and collected 8,210 interviews. Among those respondents, 83% were interviewed also in the first wave (6,811 cases). The analyses employed to test the hypotheses mostly refer to data coming from the first wave, but we also employed the two-wave panel component for further longitudinal analysis concerning H1a and H1b.

### Measures

#### Dependent variables

The dependent variable of the study is the willingness to accept freedom restrictions. We measured it by computing an additive index of two items of the same battery respectively measuring willingness to accept limitations to freedom of movement and of meeting other people (“To eliminate the diffusion of the Coronavirus, how much willing are you to limit the following personal freedom? a. Freedom of movement; b. Freedom to meet whoever you want”). Both the original items were measured on a 0–10 scale where 0 means not at all willing and 10 means totally willing. They showed a high correlation (Pearson correlation coefficient = 0.76), corresponding to a Cronbach’s alpha equal to 0.86. The final index ranges from 0 to 10, where 0 indicates the lowest willingness to limit individual freedoms, and 10 the highest one.

#### Independent variables

One of the independent variables of the study is party preferences. Here, we derived party preferences from a battery asking for every individual her propensity to vote (PTV) for each of the five main Italian parties: Pd, M5s, *Forza Italia*, *Lega* and FdI.^2^ Every individual had to indicate her likelihood to vote for each of those parties in a hypothetical future election, on a 0–10 scale where the value 0 corresponds to 'not at all likely’ and the value 10 to ‘very likely’. The PTVs are largely employed in the electoral studies as they are non-ipsative measures—their sum is not bounded to a fixed total—and allow detecting multiple party preferences (van der Ejik et al. 2006). To the aim of our research, we built a single variable of party preference based on the five PTVs. In detail, when a respondent reported at least one PTV equal or higher than 6 and assigned the highest value of the PTVs to a single party, we assigned her party preference to that party. Instead, when a respondent reported at least two PTVs equal or higher than 6 and more than two parties received the highest PTV, we assigned the individual party preference to a category indicating the multiple party preference. In this way, it is possible to analyse attitudes of “undecided or fluctuating” voters, something that is not possible through ipsative measures such as traditional voting intentions.^3^ For the purposes of our article, this allows to measure attitudes towards freedom restrictions of respondents who have not a clear preference for a single party, but still have multiple preferences for parties falling on the same side either of the ideological dimension or of the government-opposition divide. Finally, a residual category included all the respondents whose propensity to vote for each party was lower than 6. Overall, as regards their party preferences, respondents are classified as follows: Pd, M5s, *Forza Italia*, *Lega*, FdI, Pd/M5s (those who reported the same PTV equal or higher than 6 for Pd and M5s and lower PTVs for the other parties), M5s/right-wing parties (those who reported the same PTV equal or higher than 6 for M5s and at least one right-wing party, and a lower PTV for Pd), right-wing parties (those who reported the same PTV, equal or higher than 6, for at least two right-wing parties, and lower PTVs for Pd and M5s), none/do not know/do not answer. Respondents who reported the highest PTV, equal or higher than 6, for at least two parties, and are not included in the mentioned categories, are excluded from the analysis because of the inconsistency of their multiple party preferences and their low numerosity. In the empirical analyses, the relationship between such a measure of party preferences and willingness to accept freedom limitations allows testing both H1a and H1b. As regards H1a, indeed, we can consider as “progressive” voters those who prefer Pd and (at least partially) those who are undecided between Pd and M5s. Similarly, we can consider as “conservative” voters the categories *Forza Italia*, *Lega*, FdI, right-wing parties and (at least partially) M5s/right-wing parties. As far as H1b is concerned, we can consider as supporters of government parties the categories Pd, M5s and Pd/M5s, whereas we can consider as supporters of opposition parties the categories *Forza Italia*, *Lega*, FdI, right-wing parties. As we will explain later, H1b will also be tested longitudinally through panel data by considering that two opposition parties (*Forza Italia* and *Lega*) became government parties (see Sect. 2).

According to our hypotheses (H2 and H3), the other two independent variables are confidence in institutions and collectivistic orientations. Institutional trust is measured by trust in the national parliament, which can be more specifically intended as an indicator of trust in political institutions and ranges from 0 (no trust at all) to 10 (complete trust).^4^

Collectivistic orientations are measured by the additive index of two items whose wording come from the East-Asian Barometer and respectively reads as follows:

- “In a group, we should sacrifice our individual interests for the sake of the group’s collective interest”.
- “For the sake of national interest, the individual interest could be sacrificed”.

Both the items were measured on a 0–10 scale, where 0 corresponds to totally disagree and 10 to totally agree. The correlation between the two items is equal to 0.61, and accordingly the value of Cronbach’s alpha is 0.75. Moreover, previous research on the Asian context has shown that the items measure the same underlying construct (Yin 2018). The index was measured on the same scale of the two items, 0–10, where 0 indicates the highest level of individualistic orientations and 10 the highest level of collectivistic orientations.

#### Control variables

To account for possible antecedent variables in the relationship between the independent variables and the willingness to accept freedom limitations, in the empirical analysis we included the following control variables: sex, level of education (low, medium, high), age class (18–34, 35–54, 55 and more), occupational status (employed in the public sector, employed in the private sector, self-employed, unemployed, inactive), area of residence (a simplified version of geopolitical areas: North, former Red Zone, Centre-South and Islands).^5^

### Methods

Research hypotheses were tested by using multilevel linear regression models, where respondents are nested into the day of interview. Multilevel models allowed accounting for the non-independence of interviews over time, as time-varying characteristics of the context could have an impact on the dependent variable.

We first tested H1a and H1b by including in the multilevel regression model only party preferences as independent variables, in addition to the control variables (Model 1). Then, we tested the same hypotheses controlling for trust in parliament and collectivistic orientations, which were added in Model 2. The same model also allowed testing both H2 and H3. Finally, to test the moderating role of trust in parliament (H4) and collectivistic orientations (H5) on the relationship between party preferences and willingness to accept freedom restrictions, we added an interaction term between trust in parliament and party preferences (Model 3), and between collectivistic orientations and party preferences (Model 4).^6^

To further test H1a and H1b, we also employed longitudinal panel data, which allowed properly distinguishing between the ideological and the government-opposition explanation as regards the relationship between party preferences and attitudes towards freedom limitations. As explained in Sect. 2, in 2021 a new cabinet led by Mario Draghi settled down in Italy. All the parties except for FdI supported the government. The presence of right-wing parties both among government and opposition parties (on the former side, *Forza Italia* and *Lega*, on the latter side, FdI) offered the possibility of disentangling the ideological and the government-opposition explanation by using two-wave panel data. Panel data allowed analysing the individual variation in attitudes towards freedom limitations between the two waves by party preferences. To avoid endogeneity issues between the variation in party preferences and the variation in attitudes towards freedom limitations, we considered in the analysis only those individuals providing the same party preferences in both the waves. Moreover, we excluded from the analyses those people providing multiple party preferences, because of the impossibility of distinguishing between government and opposition parties.^7^ Accordingly, the analysis was performed on a subsample of the panel component, that included 2,398 respondents. To test our hypotheses, we analysed the variation in attitudes towards freedom limitations by party preferences (stable from 2020 to 2021, five categories: Pd, M5s, *Forza Italia*, *Lega*, FdI) by controlling for all the socio-demographic variables included in Models 1–4 and measured in wave 1 and for the individual variation of trust in parliament and collectivistic orientations^8^ between the two waves. Analogously to the analyses on cross-sectional data, we employed multilevel linear regression, where individuals were nested into days of the wave-1 interview, to account for the day of the interview in wave 1.

## Findings

**Fig. 1:**
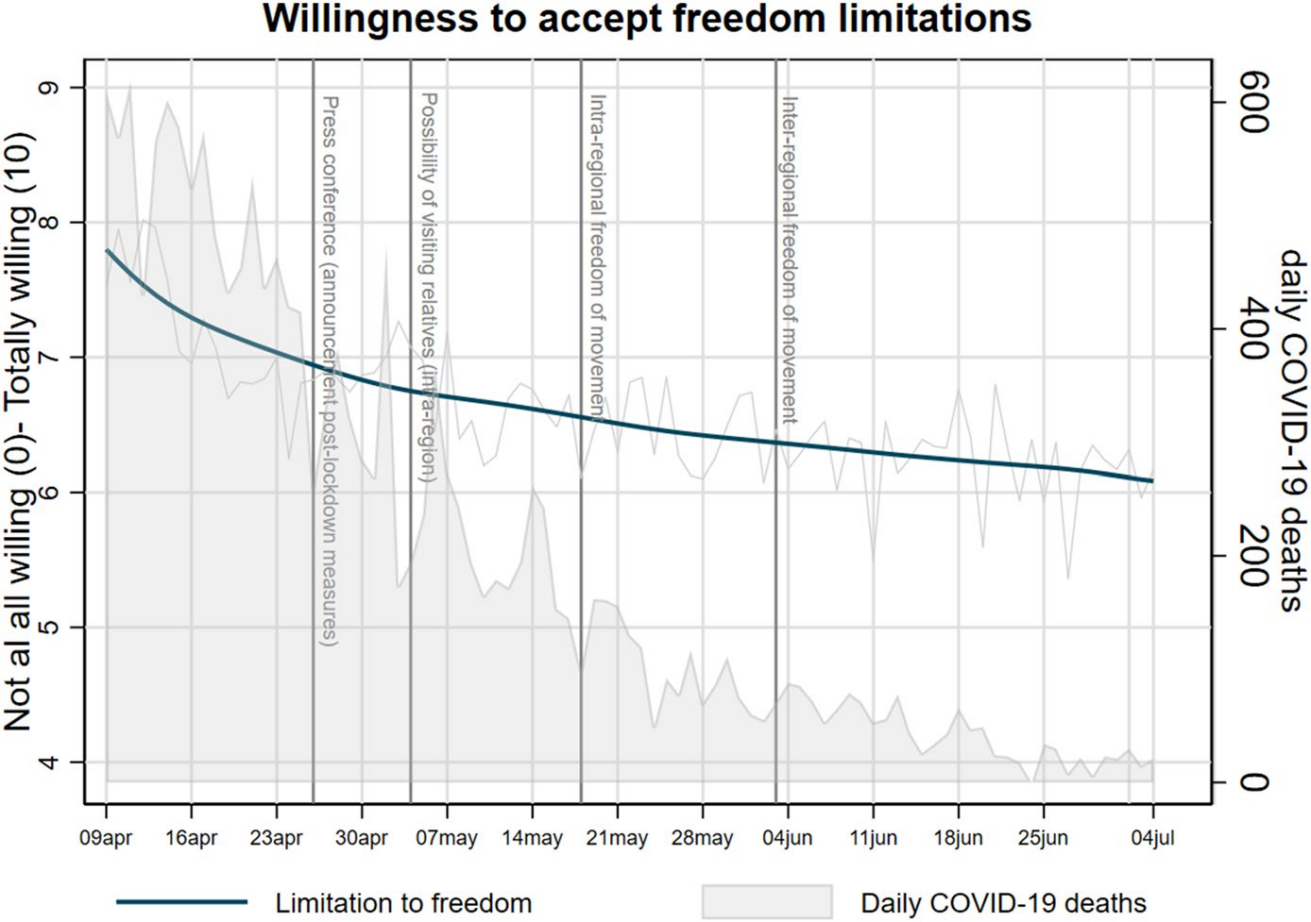
LOWESS estimations (bandwidth = 0.6) of daily means of willingness to accept freedom limitations (n = 14,260) and daily frequency of Covid-19 deaths. ResPOnsE COVID-19 wave-1 data, April 9–July 4, 2020

Before testing our hypotheses, we provide empirical evidence to answer the first research question presented in the introductory section, namely, whether and how attitudes towards freedom limitations have varied across the timespan of the first wave of the survey, in the light of the evolution of the pandemic and the changing measures taken by the Italian government. Such empirical evidence also allows better contextualizing survey data within the pandemic period, which has been highly dynamic. The rolling cross-section design allows guaranteeing a fine granularity of the observations over the timespan and, accordingly, detecting the impact of contextual characteristics on the dynamics of public opinion. In Fig. 1, we show both the LOWESS estimations of the mean of the index measuring attitudes towards freedom limitations^9^ and the official number of daily Covid-19 deaths, intended as an indicator of the intensity of the pandemic, both referred to the timespan between April 9 and July 4, 2020.^10^ Figure 1 also highlights the dates of the most significant political events and policy measures during the period of observation: April 26, the day of a press conference of the Prime Minister Giuseppe Conte who announced the calendar of post-lockdown measures; May 4, the day of the beginning of a new phase after the strict lockdown, in which people were allowed to visit their parents; May 18, the date in which free intra-regional movement was allowed and a lot of economic activities reopened; June 3, the beginning of a phase in which the free inter-regional movement was allowed and several restrictions were relaxed.

Figure 1 shows that, overall, Italian respondents were rather willing to accept freedom limitations, as the average of the index (measured on a 0–10 scale in which higher values correspond to higher willingness) is higher than 6 across the whole timespan. Nonetheless, such willingness shows a progressive decrease over time, as its average was equal to 7.8 at the beginning of April 2020 and went down to 6.1 after three months. Especially, we observe a substantial decrease across the first fifteen days of the fieldwork, during a period of strict lockdown in which the intensity of the pandemic was diminishing. Overall, as long as the pandemic risks were perceived as lower in light of the decreasing intensity of the pandemic, the agreement towards freedom restrictions was decreasing as well. Although descriptive results do not allow clearly distinguishing the effect of the intensity of the pandemic from the effect of the political events and the implementation of new restrictive policies, we do not find substantial over-time variation in the acceptance of freedom restrictions after the introduction of those policies. Therefore, Fig. 1 preliminarily suggests that the dynamics of public opinion during Covid-19 pandemic was more influenced by the changing intensity of the pandemic than by the changing policy measures (see similar evidence in Schraff 2020). Furthermore, although since June 15 several containment measures were further relaxed, the willingness to accept freedom restrictions was still declining, as respondents were plausibly less threatened by the pandemic.

The substantial over-time variation of the distribution of the dependent variable, shown in Fig. 1, is a further element supporting the use of multilevel models, which account for across time variability, to test our research hypotheses. In this regard, Model 1 in Table 1 allows preliminary testing H1a and H1b (competing hypotheses on the relationship between party preferences and attitudes towards freedom limitations). Among control variables, women show a substantially higher willingness to accept freedom restrictions than men, net of party preferences and other socio-demographic characteristics. Also, individuals living in the centre-southern regions and in the islands report a higher agreement towards freedom limitations than those living in northern regions and in the former Red Zone. Then, people aged 55 and more are slightly more willing to accept freedom limitations. While educational level proves not to be associated with the willingness to limit personal freedom, self-employed and unemployed people (on average the most hit by the negative economic consequences of the restrictive measures enacted to counteract the pandemic) show (not surprisingly) a lower willingness.

**Table 1:** Multilevel linear regression models for the estimation of attitudes towards freedom restrictions. ResPOnsE COVID-19 wave-1 data.

Independent variables	Categories	Model 1	Model 2
Gender (Ref. cat.: Male)	Female	0.49***	0.50***
		(0.05)	(0.04)
Age class	35–54	0.05	0.07
(Ref. cat.: 18–34)		(0.06)	(0.06)
	55 and more	0.17***	0.07
		(0.06)	(0.06)
Educational level	Medium	− 0.02	− 0.04
(Ref. cat.: Low)		(0.08)	(0.08)
	High	− 0.07	− 0.11
		(0.09)	(0.08)
Area of residence	Red area	0.02	− 0.02
(Ref.cat.: North)		(0.07)	(0.06)
	South and Islands	0.23***	0.14***
		(0.05)	(0.05)
Occupational status	Private sector	− 0.06	0.03
(Ref. cat.: Public sector)		(0.07)	(0.07)
	Self-employed	− 0.27***	− 0.10
		(0.09)	(0.08)
	Unemployed	− 0.39***	− 0.15
		(0.10)	(0.09)
	Other	− 0.09	− 0.01
		(0.07)	(0.07)
Party preference	Pd-M5s	− 0.24	− 0.20
(Ref. cat: Pd)		(0.17)	(0.16)
	M5s	− 0.17**	− 0.04
		(0.08)	(0.07)
	M5S/right- wing party	− 0.68***	− 0.63***
		(0.20)	(0.18)
	Forza Italia	− 0.50***	− 0.24*
		(0.13)	(0.12)
	Lega	− 0.98***	− 0.17**
		(0.09)	(0.09)
	FdI	− 1.07***	− 0.39***
		(0.09)	(0.09)
	2–3 right-wing parties	− 1.10***	− 0.31***
		(0.10)	(0.10)
	None/DK/DA	− 0.89***	− 0.29***
		(0.06)	(0.06)
Trust in parliament	0–10		0.16***
			(0.01)
Collectivistic orientations	0–10		0.35***
			(0.01)
Constant		6.86***	3.72***
		(0.14)	(0.14)
Variance (Individual level)		2.67	2.50
Variance (Day level)		(0.02) 0.46 (0.04)	(0.02) 0.38 (0.04)
Observations		13,944	13,944
Days		95	95

Standard errors in parentheses

***p < 0.01, **p < 0.05, *p < 0.1

When analysing the relationship between party preferences and attitudes towards freedom limitations, results of Model 1 in Table 1 provide empirical evidence in favour of H1b and against H1a. As regards the latter, individuals preferring Pd, the main Italian centre-left party, report the highest level of willingness to accept freedom limitations. People preferring M5s and both Pd and M5s are only slightly less willing than those preferring Pd: on average, a predicted difference respectively of − 0.17 and − 0.24 (the latter not statistically significant). Conversely, individuals who prefer any right-wing party are significantly and substantially less likely to accept freedom limitations, when compared with individuals preferring Pd. More specifically, the difference is lower for those preferring *Forza Italia* (− 0.50)—the most moderate of the three right-wing parties—or both M5s and at least one right-wing party (-0.68), whereas such difference is (similarly) higher for those preferring *Lega* (− 0.98), FdI (− 1.07) or at least two right-wing parties (− 1.10). This preliminary evidence suggests that the relationship between party preferences and acceptance of freedom limitations could be reversed from what was expected according to the classical *ideological explanation* (H1a): rather than “conservative” people, “progressive” people are more likely to accept freedom limitations to counteract the pandemic. Instead, it supports the *government-opposition explanation* (H1b) as individuals preferring parties supporting Giuseppe Conte’s government—Pd and M5s—were more likely to accept freedom limitations during the pandemic.

However, the relationship between party preferences and attitudes towards freedom limitations could be explained by other attitudes and orientations, such as trust in parliament and collectivistic orientations. After controlling for those variables (see Model 2 in Table 1), the differences in the willingness to accept restrictions across party preferences are largely reduced, especially the differences between supporters of right-wing parties and supporters of other parties. While the average difference between supporters of Pd and supporters of M5s gets close to zero, and the one between people preferring Pd and people preferring both Pd and M5s remains not statistically significant, the differences between people preferring Pd and those preferring any right-wing party remain statistically significant (at the p < 0.10 level for what concerns *Forza Italia*). Average differences with respect to individuals preferring Pd are equal to − 0.24 for *Forza Italia* supporters, − 0.17 for *Lega* supporters and − 0.39 for respondents preferring FdI, and to − 0.31 for those supporting at least two right-wing parties. Moreover, the same average difference is equal to − 0.63 for respondents preferring both M5s and at least one right-wing party. Although both institutional trust and collectivistic values largely explain the association between party preferences and attitudes towards freedom limitations, also net of those two variables we provided empirical evidence for the *government-opposition explanation* (H1b) and reversed evidence for the *ideological explanation* (H1a).

Model 2 also allows confirming both H2 and H3. Net of party preferences, collectivistic orientations and socio-demographic controls, trust in parliament is positively associated with attitudes towards freedom limitations (regression coefficient equal to 0.16, significant at the p < 0.001 level): the average difference in those attitudes between individuals with the highest and the lowest level of trust in parliament is equal to 1.56. The intensity of the association between collectivistic orientations and acceptance of freedom limitations is even stronger (regression coefficient equal to 0.35, significant at the p < 0.001 level): among people with the highest level of collectivistic orientations, the average of the index measuring the willingness to accept freedom limitations is 3.48 higher than that found among people with the highest level of individualistic orientations.

Overall, empirical results suggest that opinions towards freedom limitations largely depend on institutional trust and, even to a greater extent, on collectivistic orientations. Moreover, when compared to the effect of those two predictors, the effect of party preferences seems to be weaker. To test H4 and H5, we analyse whether the differences in the willingness to accept freedom limitations among people with different party preferences are higher when people show low trust in institutions (H4) and individualistic values (H5). In other words, we expect that when individuals report high trust in institutions and high collectivistic orientations—especially in the extraordinary situation of the pandemic, these attitudes make them accept freedom limitations, irrespective of their party preference.

**Fig. 2:**
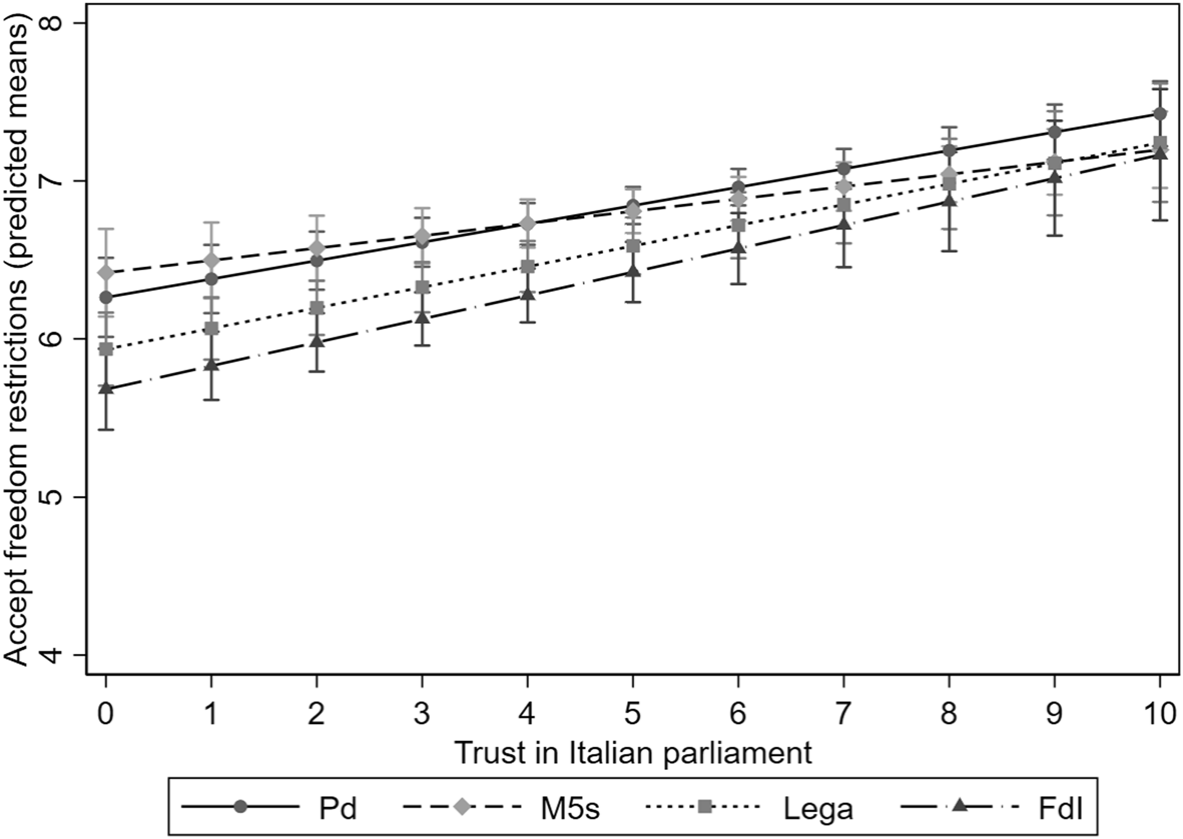
Predicted averages of attitudes towards freedom restrictions by party preferences and trust in parliament, estimated by Model 3 in Table A2 in the Online Appendix. ResPOnsE COVID-19 wave-1 data, 95% confidence intervals

Figure 2 shows the predicted averages of the index of attitudes towards freedom limitations by party preference and trust in the parliament. For the sake of clarity in the interpretation of the findings, Fig. 2 only shows the results for individuals respectively preferring the four main Italian parties: Pd, M5s, *Lega*, FdI. Notwithstanding, the whole model including all the interaction terms is shown in Model 3 in Table A2 in the Online Appendix. First, we can notice that people having high trust in parliament are more willing to accept freedom limitations, regardless of their party preferences. When looking at the moderating effect of institutional trust on the relationship between party preferences and attitudes towards freedom restrictions, results only partially validate H4. If we consider the differences in those attitudes between people preferring FdI and those preferring Pd and M5s, Fig. 2 shows a reduction of those differences corresponding to higher trust in parliament: among people with the lowest trust in parliament, the predicted value of the index is respectively 0.61 and 0.78 higher among those preferring Pd and M5s when compared with those preferring FdI; the same difference is respectively equal to 0.23 and 0.00 when people show the highest trust in parliament.^11^ Instead, when looking at people preferring *Lega*, the other right-wing populist party, the predicted difference in the dependent variable does not substantially vary by trust in the parliament. Overall, Fig. 2 shows that differences in the dependent variable only slightly increase among people with different party preferences as long as trust in parliament gets lower, but such a pattern is only partially supported by statistical significance.

**Fig. 3:**
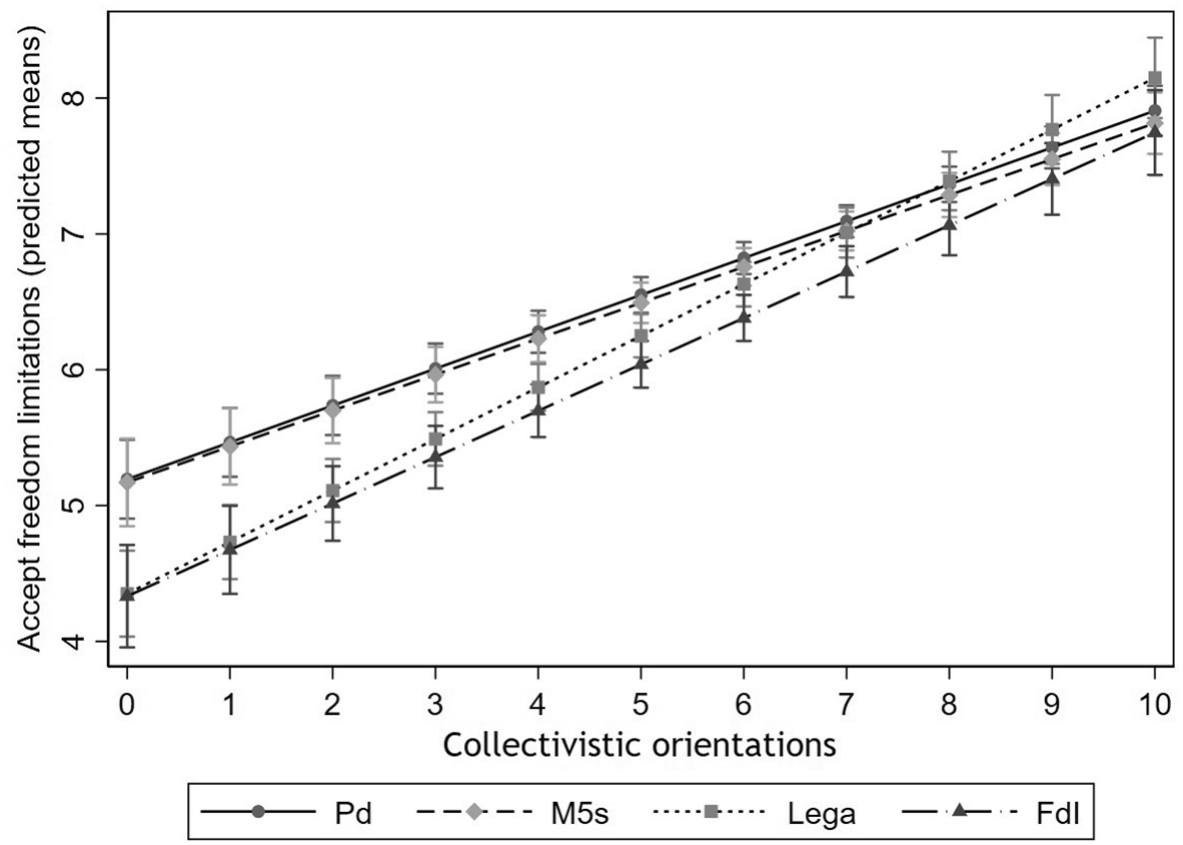
Predicted averages of attitudes towards freedom restrictions by party preferences and collectivistic orientations, estimated by Model 4 in Table A2 in the Online Appendix. ResPOnsE COVID-19 wave-1 data, 95% confidence intervals

Figure 3 shows the predicted averages of the index of attitudes towards freedom limitations by party preference (for Pd, M5s, *Lega* and FdI, as in Fig. 2) and collectivistic orientations, estimated from Model 4 in Table A2 in the Online Appendix. When people report high individualistic orientations, they always show a substantially lower acceptance of freedom limitations than those having high collectivistic orientations, regardless of their party preference. For instance, the predicted average of the index of attitudes towards freedom restrictions is equal to 5.19 among people preferring Pd with the lowest value of collectivistic orientations, while it is equal to 8.14 among people preferring *Lega* with the highest value of collectivistic orientations. However, for what concerns our hypothesis, the association between party preferences and attitudes towards freedom limitations is found only among people with lower collectivistic orientations (as expected). When people report the lowest level of collectivistic orientation (value 0), the average acceptance of freedom limitations is similar between individuals preferring Pd and those preferring M5s, as well as between people preferring *Lega* and those preferring FdI. However, the predicted index is about 0.8 higher for people preferring Pd and M5s when compared with those preferring *Lega* and FdI. Moreover, for lower levels of collectivistic orientations the confidence intervals of people supporting Pd, as well as M5s, never overlap the ones of people supporting *Lega* or FdI. In line with our expectation, people’s reported attitudes towards freedom limitations are similar among people with high collectivistic values, regardless of their party preferences. Therefore, the analysis provides strong empirical evidence supporting H5.

### Further insights on ideological and government-opposition explanation: longitudinal evidence

**Fig. 4:**
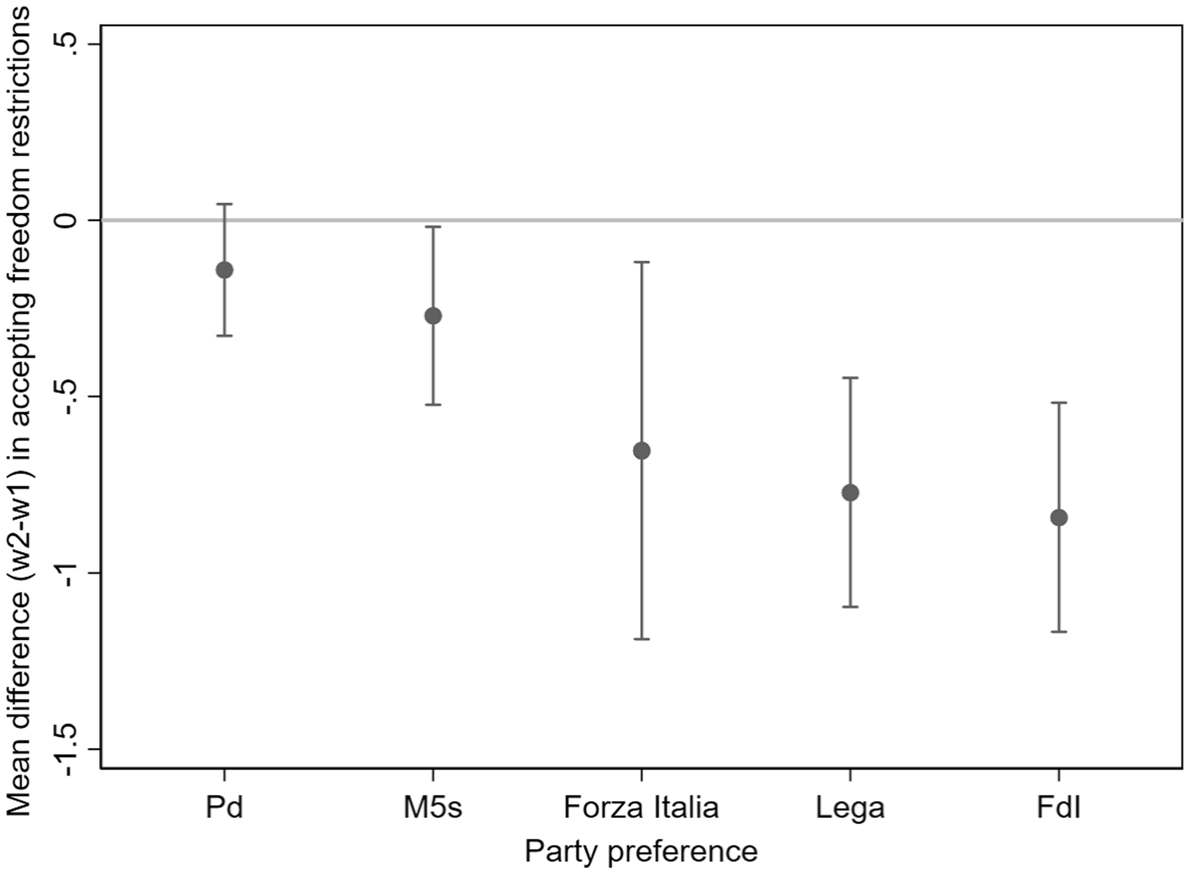
Predicted averages of individual variation in attitudes towards freedom limitations between panel wave-2 and wave-1 by party preferences, estimated by multilevel model shown in Table 2 (n = 2,398)

Previous analyses shown in Table 1 seem to support the *government-opposition explanation* (H1b) and not the *ideological explanation* (H1a) for the relationship between party preferences and attitudes towards freedom limitations. Moreover, Fig. 3 shows that people supporting right-wing opposition parties are more likely to accept freedom restrictions than other people only when they have individualistic orientations. Nonetheless, such analyses do not rule out an alternative explanation to the government-opposition one, namely, a “reversed” ideological explanation that will be thoroughly discussed in the concluding section, according to which right-wing supporters are less likely to accept freedom limitations. Thanks to the use of longitudinal panel data, we are able to provide further insights on such possible explanations. The average of the individual variation in attitudes towards freedom limitations between wave-2 (2021) and wave-1 (2020) is equal to − 0.39 on the whole panel sample (n = 6630). Since *Forza Italia* and *Lega* are opposition parties in 2020 and government parties in 2021, while FdI was always at the opposition, we should expect, according to H1b, a more negative variation in attitudes towards freedom limitations among people preferring FdI when compared to people preferring *Forza Italia* and *Lega*. In particular, we should focus on the over-time differences between people supporting respectively *Lega* and FdI, two similar right-wing populist parties. Figure 4 shows the predicted averages of the individual variation in attitudes towards freedom limitations by party preferences (estimated from the multilevel linear regression model shown in Table 2). The findings provide low support to the government-opposition explanation, as the negative average variations in attitudes towards freedom limitations is very similar for individuals preferring *Lega* (− 0.77), FdI (− 0.85) and also *Forza Italia* (− 0.64). Moreover, all these average variations are substantially more negative than the average variation for the whole panel sample. For what concerns individuals supporting Pd and M5s, the average variation over time of attitudes towards freedom limitations is substantially lower when compared to individuals supporting centre-right parties. In detail, the average variation is not statistically lower than 0 for individuals preferring Pd, while it is slightly lower than zero (− 0.27, significant at p < 0.05) for those preferring M5s; however, as reported also in Table 2, the average variation is not significantly different between supporters of the two parties. Therefore, when considering individuals supporting government parties, the average variations over time in attitudes towards freedom restrictions prove to be largely heterogeneous and highly dependent on the party supported. Overall, empirical evidence shows that the willingness to accept freedom restrictions diminishes over time especially among people preferring right-wing parties, irrespective of preferring either a government or an opposition party.

**Table 2:** Multilevel linear regression model for the estimation of the individual variation in attitudes towards freedom limitations between panel wave-2 and wave-1

Independent variables	Categories	
Gender (Ref. cat.: Male)	Female	0.50***
		(0.04)
Age class	35–54	− 0.17
(Ref. cat.: 18–34)		(0.12)
	55 and more	0.31
		(0.19)
Educational level	Medium	0.80***
(Ref. cat.: Low)		(0.18)
	High	− 0.30
		(0.19)
Area of residence	Red Area	− 0.12
(Ref.cat.: North)		(0.21)
	South and Islands	0.24
		(0.17)
Occupational status	Private sector	− 0.03
(Ref. cat.: Public sector)		(0.13)
	Self-employed	− 0.19
		(0.19)
	Unemployed	0.18
		(0.23)
	Other	− 0.06
		(0.29)
Party preference	M5s	− 0.12
(Ref. cat: Pd)		(0.16)
	Forza Italia	− 0.50*
		(0.29)
	Lega	− 0.62***
		(0.19)
	FdI	− 0.70***
		(0.19)
Trust in parliament	− 10 to 10	0.11***
(Wave2—wave1)		(0.02)
Collectivistic orientations	− 10 to 10	0.07***
(Wave2—wave1)		(0.02)
Constant		− 0.32
		(0.32)
Variance (Individual level)		2.82***
Variance (Day level)		(0.04) 0.39*** (0.10)
Observations		2,398
Days		95

Standard errors in parentheses

***p < 0.01, *p < 0.1

Table 2 also shows that, net of the other independent variables, over-time positive variations in trust in parliament and collectivistic orientations are associated with less negative—or more positive—over-time variations in attitudes towards freedom limitations.

## Conclusions

In the last decades, modernisation processes have triggered deep changes in values characterising Western societies. For what concerns the political sphere, post-materialistic values oriented towards self-realisation and self-empowerment have been affirmed. In general, individualistic values are gradually replacing collectivistic ones in Europe and other Western countries (van Tubergen 2020). However, these processes have been challenged by the outbreak of the Covid-19. Although individualistic values are largely spread in Western societies (Welzel 2013), the existential insecurity caused by the pandemic could have led to an increase of collectivistic values in those contexts. Furthermore, public health policies aimed at counter the pandemic are based on a collectivistic logic that puts the collective interest before the individual one. To contain the infections from Covid-19, most governments of democratic countries have imposed restrictions on some individual freedoms, which entail the democratic dilemma between public health and civil liberties. Hence, we wondered how attitudes towards public measures limiting individual freedoms (especially freedom of movement and of gathering) were shaped by political preferences and value orientations, in the first western democracy severely hit by the pandemic—Italy.

In this regard, we analysed survey data aimed at monitoring public opinion on multiple issues during the COVID-19 pandemic. As the questionnaires included both items measuring traditional socio-political concepts and items specific to the COVID-19 pandemic, the measurement of some constructs (confidence in institutions, collectivistic orientations—measured by a single item in wave 2) is suboptimal. Nonetheless, the rolling-cross-section design and the panel component offered the unique possibility of dynamically assessing the relationship between well-established attitudes and COVID-19-specific attitudes.

First, our analysis shows that, during the first wave of the pandemic, Italians were rather willing to accept freedom limitations and these attitudes seem to be more influenced by the changing intensity of the pandemic than by the changing policy measures (with a decline in the agreement towards freedom restrictions when the intensity of the pandemic in terms of deaths got lower). Then, we tested the hypotheses about the individual characteristics that could influence attitudes towards freedom limitations. Relying on previous literature on situations of perceived collective threats such as terrorist attacks, we hypothesised two rival hypotheses about the effect of party preferences on attitudes towards freedom limitations, one pointing out the role of the classical ideological distinction between progressivism and conservatism and the other stressing the role of the government-opposition distinction. In particular, we expected that in the pandemic context individuals preferring right-wing parties are more likely to accept restrictions on freedom (H1a) or that individuals preferring opposition parties are less likely to accept restrictions on freedom (H1b). Then, we hypothesised that the acceptance of freedom limitations to counteract the pandemic was higher among people trusting institutions (H2) and with collectivistic values (H3). Furthermore, we expected that both confidence in institutions and collectivistic orientations could moderate the effect of party preferences on attitudes towards freedom limitations: party preferences should explain differences in those attitudes especially when people have low institutional trust (H4) and individualistic orientations (H5).

Findings from multilevel regression models have rejected H1a, not fully confirmed H1b and H4, confirmed H2, H3 and H5. As regards party preferences, neither a classical ideological explanation nor a government-opposition explanation seems to be adequate to fully account for the mechanisms behind acceptance of freedom limitations. In particular, we have found an opposite pattern to that expected according to the classical ideological explanation: people with “progressive” party preferences are more inclined to accept freedom limitations to contain Covid-19 contagions, rather than people with “conservative” party preferences. As for the government-opposition explanation, when we moved from cross-sectional analysis to longitudinal analysis, we have seen that supporters of two conservative parties (*Forza Italia* and *Lega*) that moved from opposition to government significantly decreased their willingness to accept freedom limitations imposed by the government itself and these variations over time were significantly different compared to those of Pd supporters (the main centre-left Italian party). Furthermore, we have seen that party preferences are associated with different attitudes towards freedom restrictions only when people have individualistic orientations. 

The results of this study lead to two main implications. First, this study supports the assumption that individualism generally reduces the acceptance of freedom limitations and we show that individualistic people preferring right-wing parties are much less willing to accept freedom limitations to counteract the pandemic. This finding is particularly puzzling as among supporters of right-wing populist parties (*Lega* and FdI) the willingness to accept freedom limitations does not depend on the government-opposition status of the party supported. Right-wing populism and authoritarianism are often thought to be closely linked to each other, if we look at right-wing parties’ emphasis on law and order, their opposition to cultural diversity, the scepticism towards representative forms of democracy. Furthermore, conservative voters are generally more risk averse (Crawford 2017). Hence, supporters of these parties should have been strongly in favour of draconian measures to protect the health of the nation. But this is not the case according to our data. In this regard, the role played by individualism suggests that modern right-wing populism has an ambiguous nature, containing both authoritarian and anti-authoritarian elements as highlighted by Lütjen (2021, p. 3):

Right-wing populism is both a counter reaction to enlightenment and emancipation as well as it is its spoiled but nevertheless legitimate child. Many right-wing populists today speak the language of emancipation, even though they reject many of the liberalisation efforts that were emblematic of the (left-wing) emancipatory project of the 1960s and 1970s. They have reshaped and reconfigured the idea of emancipation in a way that now suits their own political agenda.

These parties, indeed, employ an anti-establishment and anti-elitist rhetoric, claiming that the ‘independent’, ‘autonomous’ or ‘mature citizen’ needs no mediating institutions (Blühdorn and Butzlaff 2020). This mistrust towards authorities is also revealed by the success of conspiracy thinking among modern right-wing populists (Mancosu et al. 2017): they support ‘alternative truth’ as the *real* truth and ‘alternative science’ as the *real* science, thus questioning and undermining any epistemic authority (Ylä-Anttila 2018). This individualistic element of modern-day right-wing populism is worth being further investigated through comparative analyses including countries of Eastern Europe. Indeed, as pointed out by Lütjen (2021), the societal conditions (e.g. the silent revolution of the 1970s) that permitted the emergence of this kind of populism in the West are absent in Eastern Europe, where authoritarian dispositions seem more widespread and right-wing parties once in power capitalise on sometimes openly anti-democratic attitudes to dismantle the constitutional checks and balances (Duriez et al. 2005).

Finally, the second implication of this study is that collectivism is the strongest predictor of positive attitudes towards the freedom limitations enacted to counteract the pandemic. Moreover, among people with collectivistic orientations, the political differences (measured through party preferences) in accepting freedom limitations disappear. While the linkage between such value orientations and political attitudes has been largely investigated in Asian countries, to our knowledge there is limited research on this regarding Western countries. Our analysis has shown that is something worth to be further investigated by relying, again, on comparative data. Indeed, individualism and collectivism are ambiguous values, in the sense that they can be interpreted in different ways and lead to different political outcomes. Individualism can become a synonym of selfishness or can become civic and universalistic, by stimulating emancipation (Welzel 2013). For what concerns collectivism, it can join individuals in social movements to pursue emancipatory political goals or against a common enemy such as Covid-19, but there is also a form of collectivism that subordinates individuals to an authority by leveraging mechanisms of conformity. With all the risks for democracy that come with it.

## Supplementary Information

Below is the link to the electronic supplementary material.

Supplementary file1 (DOCX 29 kb)

## Data Availability

Data are currently under embargo. They will be soon available after the public release on the website https://dataverse.unimi.it/dataverse/unimi

## Footnotes

1 This project has been carrying out by the research laboratory SPS TREND "Hans Schadee" of the University of Milan. Further information about the project is provided in Sect. 3 and is also available here: https://www.spstrend.it.

2 In the last general election, which took place in March 2018, those parties obtained overall 87% of the valid votes.

3 In addition to the possibility of distinguishing between respondents who provide the same preferences to more than two parties and those who provide the highest preference to a single party, the choice of using PTVs instead of voting intentions is explained by an issue of data availability, as voting intentions were asked in the ResPOnsE COVID-19 questionnaire only from May 8, 2020, one month after the beginning of the field.

4 The questionnaire contains a battery that even includes confidence in the police, the EU, and the church. As argued in previous research (e.g. Martini and Quaranta 2019; for the Italian context Ladini 2020), those items refer to different dimensions of confidence in institutions. Nevertheless, some studies have included the two items of confidence in the parliament and the police in the same index of political trust (e.g. Marien and Hooghe 2011). Since the two items are weakly correlated (0.35) in the data employed, we have decided not to include them in a single index. For robustness check, we have run all the analyses by substituting trust in parliament with trust in the police, and the results are consistent.

5 The North consists of the following regions: Aosta Valley, Piedmont, Lombardy, Trentino-South Tyrol, Veneto, Friuli-Venezia Giulia, and Liguria. The former Red Zone is made up of Emilia-Romagna, Tuscany, Marche, and Umbria. Finally, Lazio, Abruzzo, Molise, Campania, Apulia, Basilicata, Calabria, Sicily, and Sardinia are included in the Centre-South and Islands.

6 Summary statistics for all the variables included in the models are shown in Table A1 in the Online Appendix.

7 This applies especially to the categories ‘right-wing parties’ and ‘M5s/right-wing parties’. We also excluded individuals with stable multiple preferences for both Pd and M5s because of the low numerosity.

8 Collectivistic orientations were measured in this analysis by a single item (“In a group, we should sacrifice our individual interests for the sake of the group’s collective interest”), as the other (“For the sake of national interest, the individual interest could be sacrificed”) was not asked in wave 2.

9 Post-stratification weights—based on the cross-classification of gender, area of residence, age group and level of education—were applied to the LOWESS estimations shown in Fig. 1. Multilevel multivariate models all control for these variables.

10 Because of sampling issues, the first three days and the last four days are not considered in these dynamic analyses.

11 All these values refer to differences between the predicted averages shown in Fig. 2 and computed from Model 3 in Table A2 in the Online Appendix.
