# Supplementary material for: The role of party preferences in explaining acceptance of freedom restrictions in a pandemic context: the Italian case
Source: Qual Quant. 2022 Jun 7;57(Suppl 1):99–123. doi: 10.1007/s11135-022-01436-3 (PMC9170558; doi:10.1007/s11135-022-01436-3)
Supplement: Supplementary file 1 — Supplementary file1 (DOCX 29 kb) [file 11135_2022_1436_MOESM1_ESM.docx]

**Supplementary Materials**

**Table A1** Summary statistics of the variables employed in multilevel regression analysis with wave-1 data. N= 13,944. Post stratification weights are applied

|  |  |  |  |
| --- | --- | --- | --- |
| Variables | Categories/Range | Mean/Proportion | S.D. |
| *Dependent variable*  Willingness of accepting freedom limitations  *Independent variables* | 0-10 | 6.55 | 2.79 |
| Gender | Female | 47.81 |  |
|  | Male | 52.19 |  |
| Age class | 18-34  35-54 | 21.92  37.38 |  |
|  | 55 and more | 40.69 |  |
| Educational level | Low | 33.74 |  |
|  | Medium | 45.94 |  |
|  | High | 20.31 |  |
| Area of residence | North | 40.04 |  |
|  | Red Area | 16.82 |  |
|  | South and Islands | 43.14 |  |
| Occupational status | Public sector | 11.96 |  |
|  | Private sector | 30.22 |  |
|  | Self-employed | 9.99 |  |
|  | Unemployed | 9.10 |  |
|  | Other | 38.73 |  |
| Party preference | Pd | 22.14 |  |
|  | Pd-M5s | 1.76 |  |
|  | M5s | 15.68 |  |
|  | M5s/right-wing party | 1.63 |  |
|  | Forza Italia | 3.59 |  |
|  | Lega | 11.80 |  |
|  | FdI | 7.91 |  |
|  | 2-3 right-wing parties | 6.97 |  |
|  | None/DK/DA | 28.52 |  |
| Trust in parliament | 0-10 | 4.53 | 2.73 |
| Collectivistic orientations | 0-10 | 5.81 | 2.42 |

**Table A2**. Multilevel linear regression models for the estimation of attitudes towards freedom restrictions (Models 3 and 4 with interaction terms). ResPOnsE COVID-19 wave-1 data.

| Independent variables | Categories | Model 3 | Model 4 |
| --- | --- | --- | --- |
|  |  |  |  |
| Gender (Ref. cat.: Male) | Female | 0.51*** | 0.51*** |
|  |  | (0.04) | (0.04) |
| Age class | 35-54 | 0.07 | 0.07 |
| (Ref. cat.: 18-34) |  | (0.06) | (0.06) |
|  | 55 and more | 0.07 | 0.08 |
|  |  | (0.06) | (0.06) |
| Educational level | Medium | -0.03 | -0.04 |
| (Ref. cat.: Low) |  | (0.08) | (0.08) |
|  | High | -0.11 | -0.12 |
|  |  | (0.08) | (0.08) |
| Area of residence | Red Area | -0.02 | -0.03 |
| (Ref.cat.: North) |  | (0.06) | (0.06) |
|  | South and Islands | 0.14*** | 0.14*** |
|  |  | (0.05) | (0.05) |
| Occupational status | Private sector | 0.03 | 0.03 |
| (Ref. cat.: Public sector) |  | (0.07) | (0.07) |
|  | Self-employed | -0.10 | -0.09 |
|  |  | (0.08) | (0.08) |
|  | Unemployed | -0.15 | -0.15 |
|  |  | (0.09) | (0.09) |
|  | Other | -0.02 | -0.01 |
|  |  | (0.07) | (0.07) |
| Party preference | Pd-M5s | 0.44 | -0.16 |
| (Ref. cat: Pd) |  | (0.45) | (0.55) |
|  | M5s | 0.17 | -0.02 |
|  |  | (0.18) | (0.21) |
|  | M5S/right-wing party | -2.13*** | -2.13*** |
|  |  | (0.54) | (0.64) |
|  | Forza Italia | -0.25 | -0.65* |
|  |  | (0.27) | (0.36) |
|  | Lega | -0.30* | -0.84*** |
|  |  | (0.16) | (0.21) |
|  | FdI | -0.61*** | -0.86*** |
|  |  | (0.17) | (0.24) |
|  | 2-3 right-wing parties | -0.83*** | -0.92*** |
|  |  | (0.18) | (0.24) |
|  | None/DK/DA | -0.74*** | -1.20*** |
|  |  | (0.14) | (0.17) |
| Trust in parliament | 0-10 | 0.12*** | 0.15*** |
|  |  | (0.02) | (0.01) |
| Collectivistic orientations | 0-10 | 0.35*** | 0.27*** |
|  |  | (0.01) | (0.02) |
| Party preference (Ref. cat: | M5s | -0.04 |  |
| Pd)*Trust in parliament |  | (0.03) |  |
|  | Forza Italia | -0.00 |  |
|  |  | (0.05) |  |
|  | Lega | 0.01 |  |
|  |  | (0.03) |  |
|  | FdI | 0.04 |  |
|  |  | (0.04) |  |
|  | PD-M5s | -0.11 |  |
|  |  | (0.08) |  |
|  | M5s/Right-wing party | 0.26*** |  |
|  |  | (0.09) |  |
|  | 2-3 right-wing parties | 0.13*** |  |
|  |  | (0.04) |  |
|  | None/DK/DA | 0.10*** |  |
|  |  | (0.03) |  |
| Party preference (Ref. cat: | M5s |  | -0.01 |
| Pd)*Collectivistic orientations |  |  | (0.03) |
|  | Forza Italia |  | 0.06 |
|  |  |  | (0.06) |
|  | Lega |  | 0.11*** |
|  |  |  | (0.03) |
|  | FdI |  | 0.07* |
|  |  |  | (0.04) |
|  | PD-M5s |  | -0.01 |
|  |  |  | (0.08) |
|  | M5S/Right-wing party |  | 0.24** |
|  |  |  | (0.10) |
|  | 2-3 right-wing parties |  | 0.09** |
|  |  |  | (0.04) |
|  | None/DK/DA |  | 0.15*** |
|  |  |  | (0.03) |
| Constant |  | 3.95*** | 4.23*** |
|  |  | (0.18) | (0.19) |
|  |  |  |  |
| Variance (Individual level) |  | 2.49 | 2.49 |
| Variance (Day level) |  | (0.01)  0.39  (0.04) | (0.01)  0.39  (0.04) |
| Observations |  | 13,944 | 13,944 |
| Days |  | 95 | 95 |

Standard errors in parentheses

*** p<0.01, ** p<0.05, * p<0.1
